# Insights into Adherence among a Cohort of Adolescents Aged 12–20 Years in South Africa: Reported Barriers to Antiretroviral Treatment

**DOI:** 10.1155/2016/4161738

**Published:** 2016-10-27

**Authors:** Mhairi Maskew, Matthew P. Fox, Denise Evans, Darshini Govindasamy, Lise Jamieson, Given Malete, Constance Mongwenyana, Karl Technau

**Affiliations:** ^1^Health Economics and Epidemiology Research Office, Department of Internal Medicine, School of Clinical Medicine, Faculty of Health Sciences, University of the Witwatersrand, Johannesburg, South Africa; ^2^Department of Global Health, Boston University School of Public Health, Boston, MA, USA; ^3^Department of Epidemiology, Boston University School of Public Health, Boston, MA, USA; ^4^Health Systems Research Unit, South African Medical Research Council, Cape Town, South Africa; ^5^Empilweni Services and Research Unit, Department of Paediatrics and Child Health, University of the Witwatersrand, Rahima Moosa Mother and Child Hospital, Johannesburg, South Africa

## Abstract

Adolescents experience disproportionately high rates of poor ART outcomes compared to adults despite prolonged use of antiretroviral therapy in Southern African treatment programs, presenting a significant challenge to national attempts to meet the UNAIDS 90-90-90 targets for 2020. This cohort study among adolescents aged 12–20 years accessing ART care at two urban public-sector clinics in Johannesburg between September and November 2013 aimed to identify factors potentially associated with poor attendance at clinic visits. Patients were followed up through routine medical records to identify missed visits (failing to attend clinic within 30 days of scheduled visit date) up to 2 years after enrolment. We enrolled 126 adolescents on ART for a median of 6.3 years (IQR: 2.7–8.4). A total of 47 (38%) adolescents missed a scheduled visit within 24 months of enrolment. Older adolescents (18–20 years) were more likely to miss a visit compared to adolescents aged 12–14 years (risk ratio (RR) = 1.72; 95% CI: 1.00–2.95). Those who were identified to have difficulty in taking medication (RR = 1.57; 95% CI: 1.13–2.18) as a barrier to care were more likely to miss a visit compared to adolescents who did not. Awareness of treatment fatigue, challenges to taking ART, and caregiver difficulties is important when considering interventions to improve treatment outcomes among adolescents.

## 1. Introduction

Despite the incredible success of the large-scale public-sector provision of antiretroviral therapy (ART) to HIV-infected people in South Africa, failure to retain these patients on long-term treatment threatens to undermine the massive gains made since 2004 and remains one of the most critical obstacles to achieving the UNAIDS 90-90-90 targets for 2020 which are intended to be a key milestone towards ending the HIV epidemic by 2030 [1]. Adolescents have been identified as vulnerable populations in need of prioritizing for adherence to treatment [2, 3]. Globally, an estimated 2 million adolescents (10–19 years) are living with HIV/AIDS, with more than 80% of these from sub-Saharan Africa [4]. The escalating HIV-infected adolescent population, which is attributed both to a high incidence of horizontally acquired HIV and to long-term survival following perinatally acquired HIV infection [5, 6], means we can expect to see large impacts of this age group on national HIV treatment programs in the coming years. The recognition of the particularly high risk faced by adolescent girls and young women has seen initiatives such as PEPFAR's DREAMS launch pilot projects involving evidence-based interventions intended to break the cycles of HIV transmission in this group [7].

Adolescence is a stage of transition from childhood to adulthood, associated with specific challenges (including puberty) and vulnerability (such as early sexual debut, HIV, and STI acquisition). In addition, coping with the clinical and psychosocial impacts of HIV imposes a substantial burden on this vulnerable group [8–11]. HIV-infected adolescents encounter several known economic barriers to accessing HIV care such as cost of transport and distance to health facility [12–14] and are often caught between pediatric and adult services unable to address their special health needs which include disclosure of HIV status, adherence support, stigma and discrimination, sexual and reproductive health, mental health care, and legal and social support [5, 15–17].

Scale-up of ART in South Africa over the past ten years has been impressive with an estimated 3.4 million HIV-infected individuals on ART by 2015 [18, 19]. However, ART coverage for children in the region has historically been considerably lower among children compared to adults (22% versus 55%, resp., in 2010) [20] though coverage among children has improved with USAID 2014 estimates indicating that nearly 50% of children <15 years of age living with HIV in South Africa are receiving ART [21]. Retention to ART is one of the universal goals of HIV treatment programs, yet there are several indications that adolescents are experiencing disproportionately poor ART outcomes compared to adults including higher rates of mortality, loss to follow-up, and lower rates of virologic suppression compared to other age categories [22–27], raising concern over the impact this group may have on national efforts to achieve the 90-90-90 targets.

Despite this, there are no national level estimates of adolescent retention in care or transfer across clinics and it remains unclear as to which adolescents are most likely to drop out of care in South Africa. Recent work using routine laboratory data to create a national HIV cohort has demonstrated the emergence of a youth treatment bulge, increasing numbers of adolescents aged 10 years and older accessing ART [28]. Given these figures and poor treatment outcomes experienced by this group, understanding the potential obstacles to adherence and retention in treatment programs among adolescents is urgently warranted. This study aimed to identify barriers and facilitators to remaining in and adhering to the continuum of ART care among HIV-infected adolescents in Johannesburg, South Africa. Identification of such factors could enable HIV care programs in this setting to target interventions most likely to reduce losses from ART care among adolescent populations.

## 2. Materials and Methods

### 2.1. Study Design and Sites

This prospective cohort study of HIV-infected adolescents accessing ART care was conducted at two public-sector antiretroviral treatment facilities in Johannesburg, South Africa. Both sites provide care and treatment according to the South African Department of Health National Antiretroviral Therapy Guidelines [12]. Under these guidelines, eligibility criteria for ART differed by age of the child. Children eligible for ART included: (1) all children <1 year of age; (2) children aged between 1 and 5 years with a WHO clinical stage 3 or 4 condition, a CD4 count of 25% or less, or an absolute CD4 count <750 cells/mL; and (3) children aged between 5 and 15 years with a WHO clinical stage 3 or 4 condition or CD4 < 350 cells/*µ*L. Adolescents >15 years were eligible for treatment with (1) a CD4 count <200 irrespective of clinical stage, (2) a CD4 count <350 cells/mm^3^ (in the presence of TB or pregnancy), or (3) a WHO stage 4 condition or MDR/XDR TB irrespective of CD4 count.

At the time of the study, the Empilweni Clinic [29, 30], a pediatric HIV care and treatment clinic based at the Rahima Moosa Mother and Child Hospital (RMMCH), was caring for approximately 400 adolescents aged 12–20 who were receiving (>80%) first-line antiretroviral treatment; the remainder of the group were receiving either second treatment or lamivudine-based holding regimens. Standard first-line regimens consisted of abacavir, lamivudine, and lopinavir/ritonavir for those <3 years of age or 10 kg in weight and abacavir, lamivudine, and efavirenz for those over 3 years of age and 10 kg. The Themba Lethu Clinic (adult-based facility) is an outpatient antiretroviral treatment facility based at Helen Joseph Hospital, Johannesburg, Gauteng [31]. The site's patient population is primarily adult (99% aged > 20 years) but at the time of the study ART and treatment monitoring were provided to approximately 80 adolescents. This includes a first-line regimen of tenofovir with lamivudine or emtricitabine plus either efavirenz or nevirapine.

### 2.2. Study Population

Eligible patients included HIV-infected adolescents aged between 12 and 20 years of age who were aware of their status and accessing ART treatment at either Empilweni Clinic or Themba Lethu Clinic regardless of duration of ART use. Identification of potentially eligible participants occurred during routine HIV care visits between September and November 2013 at specific clinic locations in which patients wait for relevant services (i.e., HIV counseling, doctor's visit, and pharmacy drug collection). Eligible and interested participants were provided with more detailed information about the study in a separate counseling room and individual consent (or participant assent plus parental or legal guardian consent for adolescents < 18 years of age) was obtained. This recruitment strategy proceeded in a consecutive manner until the end of the enrolment period. All study participants received a small financial compensation in the form of a food voucher and parents or legal guardians who returned to complete a scheduled study appointment with an adolescent were also reimbursed for transport money.

### 2.3. Data Sources

Trained interviewers administered a structured questionnaire to each enrolled study participant. Though most questions were closed-ended, the questionnaire also included pairwise ranking and some open-ended questions. Participant data from the questionnaire was entered into a study database using CSPro software by trained data capturers. Adolescents were asked whether they considered several factors (related to caregivers, travel to clinic, psychosocial elements, the health care facility, or the treatment itself) as potential barriers to care during the interview. Routinely collected medical record data (including clinic visit dates, ART regimen history, and viral load results) were extracted from electronic medical records maintained by each of the study sites and linked to participant questionnaire data.

### 2.4. Study Variables and Analysis

The primary outcome was defined as missing a scheduled ART drug collection visit by more than 30 days. Several factors were investigated as potential predictors of poor adherence. These included the following: (1) demographic characteristics of the participant, (2) socioeconomic features of the participant's household, (3) caregiver issues, and (4) problems with service delivery at the treatment facility.

The characteristics of the study population were summarized with descriptive statistics including simple proportions for categorical variables and medians with interquartile ranges for continuous variables. The overall frequency of each of the factors reported during the interview as being considered by the adolescent as a potential barrier to care is presented. In addition, we present these frequencies stratified by treatment facility and, by proxy, model of care (pediatric-based compared to adult-based treatment sites). Next, associations between each of the potential predictors and missing a clinic visit are estimated with crude risk ratios and 95% confidence intervals. Estimates were also adjusted for age, gender, mother as caregiver, and time on ART.

In secondary analysis, we defined prevalent unsuppressed viral load at the time of study enrolment as having a VL > 400 copies/mL within 3 months prior to and 3 months after the interview date. A log-binomial regression model was fitted in order to determine risk factors associated with prevalent unsuppressed viral load.

Previous work has demonstrated heterogeneity within age substrata of adolescent populations in terms of treatment outcomes [22, 25]. In order to evaluate differences in perceived barriers to care by stage of adolescence, the analysis of missed visits was further stratified by age category. Those aged between 12 and 17 years at the time of study enrolment were categorized as younger adolescents, while those aged 18–20 years were categorized as older adolescents.

## 3. Results

In total, 206 adolescents were screened for potential enrolment into the study. Of these, a total of 126 adolescents (61%) were enrolled in the study with a median of 6.3 years on ART (IQR 2.7–8.4): 19 adolescents from the adult-based facility (Themba Lethu clinic) and 107 from the pediatric-based facility (Empilweni Clinic). Of the remaining 80 not enrolled, 41 (51%) did not meet eligibility criteria and 18 (23%) were unable to consent, as their legal guardian was not present. Further 16 (8%) refused to participate (5 were in a hurry, 4 were not interested, and 7 had no reason given) and the remaining 5 were not enrolled for unknown reasons. Similar overall proportions were enrolled at the pediatric-based (27%) and adult-based (24%) facilities. The demographic characteristics of the enrolled study participants are summarized in Table 1. Adolescents at the adult-based care facility were older (median: 18 versus 15 years) and predominantly female (68% versus 54%) and had received fewer years of ART (median: 1.2 versus 6.8 years) than those at the pediatric-based facility. Nearly three-quarters (73%) were on first-line and the remaining quarter was on second-line regimens at the time of study enrolment.

### 3.1. Potential Barriers to Care

Overall, the factor most frequently reported during the interview as being considered a potential barrier to care was long travelling distance to the clinic (61% agreed it was a potential barrier to care), followed by the possibility that the adolescent's attendance at clinic visits would be noticed by friends or members of the school (33%), having an elderly caregiver (32%), high transport cost of the trip to the clinic visit (32%,) and long queues to wait in at the clinic (31%) (Table 2).

Though the factors most frequently reported to be potential barriers to care were the same for both the adult-based site and pediatric-based site, the relative importance differed slightly; having an elderly caregiver was reported as a potential barrier to care as frequently as long travelling distances to the clinic among predominantly older adolescents at the adult-based site, while it was the least frequently reported of the five among those attending care at the pediatric facility. Very few of the interviewed adolescents agreed that being disinterested in care or not believing that ART helps would be a barrier to care or that unfriendly health care workers presented problems in accessing and remaining in care.

### 3.2. Adherence to Scheduled Visits

Adolescents completing the questionnaire were then followed up passively through routine medical record data for 24 months after study enrolment. Of the 126 adolescents enrolled, 2 did not return to their treatment clinic at all after the study interview. Among the 124 that did attend at least one clinic visit after study enrolment, 92 (74%) remained in care through to the end of 24 months of follow-up, while 11 (9%) had been lost from care and 21 (17%) had transferred to another heath care facility. Overall, during the course of the 24 months of follow-up, 38% (*n* = 47) of the study participants missed a scheduled clinic visit by 7 days or more. The median time to first missed visit was 6.7 months (IQR 3.0–11.1) and 46.8% (*n* = 22) of visits were missed within 6 months and further 34.0% (*n* = 16) within a year after study enrolment.

Several demographic factors were associated with an increased risk of missing a clinical visit after study enrolment (Table 3). In crude analyses, among both of the older adolescent categories (15–17 and 18–20), the proportions missing a clinic visit were higher compared to the group of 12–14 years (41% and 49%, resp., versus 28%). Overall, the results suggest that older adolescents (15–20 years) were more likely to miss a visit compared to those in the group of 12–14 years (risk ratio (RR) = 1.59; 95% confidence interval (CI): 0.97–2.67) and adolescents in the group of 18–20 years were at highest risk of missing a clinic visit (RR: 1.72; 95% CI: 1.00–2.95) though the estimates lacked precision and statistical significance. Adolescents with their mother as their primary caregiver were somewhat less likely to miss a visit (RR: 0.67; 95% CI: 0.43–1.06). When adolescents were asked if they agreed that certain factors could be barriers to care, those who agreed that potential barriers included having a caregiver with financial difficulty (47% versus 36%), not having enough time for clinic visits (53% versus 36%), clinic visits being noticed by friends or the school (45% versus 35%), and unfriendly health care workers (75% versus 37%) more frequently missed a visit after study enrolment than those who did not identify these as barriers to care. Adolescents who agreed that having problems in taking the medication and becoming tired of taking the medication were barriers to care were also more likely to subsequently miss a visit (RR = 1.77; 95% CI: 1.14–2.74; and RR = 1.77; 95% CI: 1.14–2.73, resp.). Estimates adjusted for age, gender, mother as caregiver, and time on ART were less precise but overall consistent with crude estimates (Table 3).

### 3.3. Prevalent Unsuppressed Viral Load

In total, 103 adolescents had a recorded viral load measurement within the defined prevalent virologic outcome window period (within 3 months prior to and 3 months after the interview date), and, of these, 32 (31%) had a VL > 400 copies/mL. The frequency of prevalent unsuppressed viral load differed by several demographic factors (Table 4). A higher proportion of those in both older adolescent categories (38% for 15–17 years and 32% for 18–20 years) experienced unsuppressed viral load at the time of study enrolment compared to those aged 12–14 years (26%). Prevalent unsuppressed viral load also differed by gender (36% males versus 27% females). Higher frequency of unsuppressed viral load was also noted among adolescents who agreed that certain factors were obstacles to accessing or remaining in care compared to those who did not agree. This included having an ill caregiver (50% versus 30%), expensive transport fees (39% versus 28%), and treatment fatigue (41% versus 28%). Provider-related factors including long queues and inconvenient clinic operating hours were not perceived as barriers among those who did not have a suppressed VL.

### 3.4. Stratification by Age Category

As noted earlier, older adolescents were more likely to miss a subsequent clinic visit after study enrolment than either of the other age categories (Table 3). To determine if perceived barriers to care differed by age group, we stratified the cohort into two categories: younger adolescents aged 12–17 (Supplementary Table A in Supplementary Material available online at http://dx.doi.org/10.1155/2016/4161738) and older adolescents aged 18–20 (Supplementary Table B). In addition, having their mother as their primary caregiver appeared to decrease the likelihood of missing a clinic visit after study enrolment (27% versus 42% for 12–17 years old and 39% versus 55% for 18–20 years old). Also greater proportions of adolescents who felt that unfriendly health care workers, having problems in taking the medication, and treatment fatigue were barriers to care missed a subsequent visit compared to those who did not agree with those statements.

Lack of services at health care facilities was also noted by both age groups but the service of importance differed; younger adolescents were more impacted by lack of peer support and counseling (57% versus 31% missed visit), while older adolescents agreeing that lack of sexual health services could pose a barrier to care were more likely to miss a subsequent visit (67% versus 47%). Certain potential barriers only appeared to impact adherence to visits in one group and not the other. Among younger adolescents, visits to the clinic being noticed by friends or individuals in the school community were associated with a higher proportion of subsequent missed visits compared to adolescents not perceiving that as a barrier to care (46% versus 27%), as was agreeing that difficulty finding time to attend visits and expensive transport fees. For older adolescents, however, agreeing with the statement that having an ill caregiver who requires care (67% versus 47%) or a caregiver with financial difficulty (71% versus 43%) was barriers to care was more common among those who subsequently missed a clinic visit compared to those who did not agree with those factors as potential barriers.

## 4. Discussion

As pediatric ART has scaled up, HIV care services, mostly delivered in secondary-level health care facilities, are becoming heavily overburdened, while increasing numbers of children are transitioning to adolescence [5]. This is further complicated by the need for skilled health care workers able to manage drug toxicities and psychosocial issues as well as the higher risk of drug resistance which may result from frequent changing of fixed-dose combinations and/or drug stock-outs and lower rates of adherence to medication [32–34].

Few studies have assessed treatment outcomes in older children and/or adolescents in this region, and those that have report disproportionately poor ART outcomes in this group compared to adults. A study investigating treatment outcomes in adolescents (9–19 years) accessing care from a community-based ART program within a periurban township in Cape Town, South Africa, showed that adolescents had significantly lower rates of virologic suppression (<400 copies/mL) (27.3%) compared to young adults (63.1%) [26]. High rates of mortality and loss to follow-up (LTFU) have also been reported among adolescents compared to adults accessing care from primary health care centers [22, 24, 26, 35]. A recent study investigating outcomes in multiple HIV cohorts from Gauteng and Mpumalanga in South Africa found that attrition in ART care occurred soon after the commencement of ART; the median time to death or loss to care was 4.7 months (IQR: 1.5–13.2) and 10.9 months (IQR: 5.0–22.7), respectively. Adolescents were more likely to be LTFU after ART initiation (hazard ratio (HR) = 1.38; 95% CI: 1.07–1.78) compared to adults, though little age difference in mortality was found [25]. Despite this clear indication of increased risk for poor treatment outcomes among adolescents, the potential causes of poor adherence to treatment are yet to be clearly identified.

We interviewed a group of 126 adolescents and aimed to uncover what factors adolescents perceived to be barriers to accessing and remaining in care. Further, we estimated associations between agreeing that a factor was a barrier to care and subsequently missing a scheduled HIV clinic visit and whether these differed between older and younger adolescents. We found that the most frequently reported perceived barriers to care were related to logistics of the actual clinic visit: time and cost involved in getting to the visit as well as duration of time spent waiting in queues. Treatment programs requiring patients to return to the clinic more frequently or travel longer distances to receive necessary drugs increase the economic burden of transport costs and the possibility of lost wages for patients, both of which are well known barriers of adherence to HIV care and treatment [13, 14]. Though our sample was restricted to an urban setting, previous research has also shown that the type of community (urban versus rural) can be a factor of adherence to HIV care and treatment due to differences in community characteristics such as density of population, distance to and availability of clinics and hospitals, and infrastructure within clinics [36, 37]. Reassuringly, very few considered not being interested in treatment, unfriendly health care workers, or distrust of health care workers as important potential barriers to care, though it must be considered that adolescent experiencing these barriers personally may not be attending visits at all.

We also found that older adolescents appear to be a key subgroup of this already vulnerable population; older adolescents were at increased risk for missing a clinic visit and were also more likely to have experienced prevalent virologic failure at the time of study enrolment compared to younger age categories. This has been demonstrated elsewhere [24, 25, 38, 39] and understanding drivers of poor adherence in this group is critical. One possibility is simply that younger age groups represent perinatally infected children who represent a group who have been on treatment for longer period of time and are more likely to be adherent to treatment. However, the reality for many adolescents accessing care in this setting is that attending a clinic visit may take up a full day requiring time off school as well as time away from other household and family responsibilities. The impact of this time pressure does appear to differ between older and younger adolescents. We note that older adolescents more frequently agreed that having an elderly caregiver presented a potential barrier to care (47%) compared to younger adolescents (33%). This may reflect social circumstances where, as adolescent ages, increased responsibilities for caring for elderly members of the family are placed on them, in which, along with increasing demands during more senior years at school or entry into the workforce, time away from school/work and home responsibilities may represent a substantial obstacle in this group. While having problems in taking the medication and treatment fatigue were identified as barriers in both younger and older adolescents who missed a visit, the proportion of older adolescents that identified unfriendly health care workers as a barrier was far greater (RR: 2.19; 95% CI: 1.52–3.14, Table 3). Though the numbers represented are too small to make strong inferences, differences in provider relationships between adult-based and pediatric-based facilities could be important and modifiable factors affecting adherence to care.

Having problems taking ART (most frequently reported as forgetting to take the medication) was identified as one of the treatment-related barriers to care by both age groups of adolescents and was associated with an increased risk of poor adherence to visits. Several interventions have been tested for feasibility and efficacy in terms of reminders to take tablets and may present an opportunity to improve a relatively easily modifiable risk factor for poor adherence among adolescents. These include mobile phone text message reminders and electronic medication monitoring devices [40, 41]. Though adolescents frequently agreed that treatment fatigue was a potential barrier to accessing and remaining in care and those who agreed with this statement were more likely to subsequently miss a visit (58% versus 33%), there was little difference in the likelihood of missing visits by time on ART (40% and 36% for those on ART < 6 years and > 6 years, resp.). It is possible that attrition due to treatment fatigue occurs earlier on and the group that would be susceptible to dropping out of care due to treatment fatigue is not accurately represented in this study.

Disclosure of HIV status is an important issue in adolescent populations: not only disclosure of status to the vertically infected child but also disclosure by the adolescents themselves to family, friends, and members of their school and community. Among the group of adolescents interviewed, >95% agreed that disclosing status to family would facilitate access to care, but only a third agreed that disclosing to school and friends would be of assistance. In fact, one of the most frequently perceived barriers to care was the fact that the frequency of HIV clinic visits would be noticed by friends or members of the school community and among the group aged 12–17; perceiving this factor as a barrier to care was associated with a 70% increased risk of subsequently missing a clinic visit, underlining the social challenges and stigma that school-going adolescents face while in ART care programs.

Our findings must be considered in light of some limitations. First, we acknowledge that, by definition, all the participants interviewed were still accessing care at the time of the study and the factors identified by this group as important determinants of accessing and staying in care may differ from those that would potentially be identified by adolescents who have already dropped out of care. Second, the small numbers enrolled limited the statistical power to detect small differences and precision of our estimates. However, the analysis presented here aimed to explore possible associations between the factors investigated and adherence to ART treatment rather than establishing causal associations and should be interpreted as such. Third, we were unable to enroll some participants <18 years of age (9% of the total screened participants) due to the fact that a legal guardian was not able to be present to sign consent. If those who were not enrolled differed systematically from those <18 years that consented, this could result in selection bias. In addition, bias may arise if the need for caregiver consent to participate in the study influenced the responses provided by the adolescents interviewed. Though we cannot rule this possibility out completely, several steps were taken during the consenting and interviewing process to prevent this, including an explanation of the procedures undertaken to ensure confidentiality and conducting the interview in a private space. Finally, we did not measure depression or stigma directly as potential barriers to care and these may contribute significantly to the perception of other factors.

## 5. Conclusions

Despite these limitations, our study results are important. First, we demonstrate the increased risk of poor adherence to care for older adolescents, highlighting this group as a key population for intervention if 90-90-90 targets are to be met. In addition, we report on several modifiable factors that may be barriers to accessing and remaining in care among adolescents and show that these differ in importance and impact between older and younger adolescents. Several different interventions to improve retention in care and adherence to treatment have been proposed including financial incentives and behavioral and facility level interventions such as peer social support and adherence clubs [35, 42–46]. Evaluation of the effectiveness and potential impact of different intervention approaches is needed within the context of each of the progressive stages of adolescence. Adherence to ART is complex, and a multifaceted approach acknowledging changing barriers to accessing and remaining in care is required when designing and implementing interventions for adolescent populations.

## Supplementary Material

The supplementary material presents the analysis of risk factors associated with having at least one late or missed visit stratified by age category. First, we estimate factors associated with the primary outcome for younger adolescents aged 12-17 years (Supplementary Table A) and second, for older adolescents aged 18-20 years (Supplementary Table B).

## Figures and Tables

**Table 1:** Demographics at study enrolment.

	All (*N* = 126)	TLC (*N* = 19)	EC (*N* = 107)
Gender; *n* (%)			
Male	55 (43.7)	6 (31.6)	49 (45.8)
Female	71 (56.3)	13 (68.4)	58 (54.2)
Age (years); median (IQR)	15 (13–18)	18 (18-19)	15 (13–17)
Age group; *n* (%)			
12–14	54 (42.9)	2 (10.5)	52 (48.6)
15–17	34 (27.0)	2 (10.5)	32 (29.9)
18–20	38 (30.2)	15 (78.9)	23 (21.5)
Nationality; *n* (%)			
South African	120 (95.2)	18 (94.7)	102 (95.3)
Foreign	6 (4.8)	1 (5.3)	5 (4.7)
Highest school level; *n* (%)			
Secondary	48 (41.4)	9 (64.3)	39 (38.2)
Primary	68 (58.6)	5 (35.7)	63 (61.8)
Dwelling type; *n* (%)			
Informal	17 (13.5)	0 (0.0)	17 (15.9)
Formal	95 (75.4)	18 (94.7)	77 (72.0)
Care facility^*∗*^	14 (11.1)	1 (5.3)	13 (12.1)
Number in household; median (IQR)	4 (3-6)	3 (2–6)	5 (3–7)
caregiver type; *n* (%)			
Mother	66 (52.4)	11 (57.9)	55 (51.4)
Granny	19 (15.1)	5 (26.3)	14 (13.1)
Aunt	20 (15.9)	1 (5.3)	19 (17.8)
Children's home	13 (10.3)	1 (5.3)	12 (11.2)
Other	8 (6.3)	1 (5.3)	7 (6.5)
Caregiver employed; *n* (%)			
Yes	79 (62.7)	13 (68.4)	66 (61.7)
No	34 (27.0)	6 (31.6)	28 (26.2)
Time on ART (years); median (IQR)	6.3 (2.7–8.4)	1.2 (0.5–1.6)	6.8 (4.9–8.5)

^*∗*^Care facility included children's home, hospice, or shelter.

**Table 2:** Frequency of reported factors, overall and by site.

	All (*N* = 126)	Adult-based facility (*N* = 19)	Pediatric-based facility (*N* = 107)
*Median number of factors (IQR)*	*4 (3–6)*	*4 (3–8)*	*4 (3–6)*

Long distance to clinic; *n* (%)	77 (61.1)	10 (52.6)	67 (62.6)
Visits noticed by friends/school; *n* (%)	41 (32.5)	7 (36.8)	34 (31.8)
Caregiver is elderly; *n* (%)	40 (31.7)	10 (52.6)	30 (28.0)
Transport fee is expensive; *n* (%)	40 (31.7)	7 (36.8)	33 (30.8)
Long waiting queues at clinic; *n* (%)	39 (31.0)	6 (31.6)	33 (30.8)
Visits noticed by family/community; *n* (%)	28 (22.2)	6 (31.6)	22 (20.6)
Treatment fatigue; *n* (%)	27 (21.4)	2 (10.5)	25 (23.4)
Having problems taking ART; *n* (%)	24 (19.0)	3 (15.8)	21 (19.6)
Inconvenient clinic operating hours; *n* (%)	21 (16.7)	0 (0.0)	21 (19.6)
Caregiver financial difficulty; *n* (%)	19 (15.1)	3 (15.8)	16 (15.0)
Not enough time for visits; *n* (%)	18 (14.3)	2 (10.5)	16 (15.0)
No sexual health services; *n* (%)	14 (11.1)	1 (5.3)	13 (12.1)
Caregiver is unsupportive; *n* (%)	13 (10.3)	3 (15.8)	10 (9.3)
Lack of peer support/counselling; *n* (%)	13 (10.3)	5 (26.3)	8 (7.5)
Caregiver changes frequently; *n* (%)	8 (6.3)	1 (5.3)	7 (6.5)
Caregiver is ill and requires care; *n* (%)	8 (6.3)	2 (10.5)	6 (5.6)
Distrust health care workers; *n* (%)	7 (5.6)	1 (5.3)	6 (5.6)
Unfriendly health care workers; *n* (%)	4 (3.2)	3 (15.8)	1 (0.9)
Disinterested in care; *n* (%)	1 (0.8)	0 (0.0)	1 (0.9)

**Table 3:** Factors associated with having at least one late or missed visit (*n* = 124).

Characteristic	*N* (%) with missed visit	Crude risk ratio (95% CI)	Adjusted risk ratio^*∗*^ (95% CI)
*Baseline and demographic factors*			
Gender			
Female	27 (39.1)	1.00	1.00
Male	20 (36.4)	0.93 (0.59–1.47)	1.27 (0.76–2.12)
Age group			
12–14 years	15 (28.3)	1.00	1.00
15–17 years	14 (41.2)	1.45 (0.81–2.62)	1.80 (0.94–3.43)
18–20 years	18 (48.6)	1.72 (1.00–2.95)	2.62 (0.89–7.67)
Highest school level			
Secondary	21 (44.7)	1.00	1.00
Primary	22 (32.8)	0.73 (0.46–1.17)	1.37 (0.69–2.72)
Dwelling type			
Formal	36 (38.3)	1.00	1.00
Care facility	5 (38.5)	1.00 (0.48–2.09)	0.82 (0.40–1.70)
Informal	6 (35.3)	0.92 (0.46–1.84)	1.41 (0.65–3.09)
Mother as caregiver			
No	27 (45.8)	1.00	1.00
Yes	20 (30.8)	0.67 (0.43–1.06)	0.66 (0.43–1.03)
Caregiver employed			
No	10 (30.3)	1.00	1.00
Yes	32 (40.5)	1.34 (0.75–2.39)	1.40 (0.79–2.48)
Time on ART			
<6 years	24 (40.0)	1.00	1.00
>6 years	23 (35.9)	0.90 (0.57–1.41)	0.98 (0.42–2.29)
*Caregiver-related factors*			
Caregiver changes frequently			
No	45 (38.8)	1.00	1.00
Yes	2 (25.0)	0.64 (0.19–2.19)	0.57 (0.17–1.88)
Caregiver is elderly			
No	30 (35.7)	1.00	1.00
Yes	17 (42.5)	1.19 (0.75–1.89)	1.00 (0.63–1.60)
Caregiver is ill, requires care			
No	44 (37.9)	1.00	1.00
Yes	3 (37.5)	0.99 (0.39–2.49)	0.94 (0.37–2.38)
Caregiver is unsupportive			
No	42 (37.8)	1.00	1.00
Yes	5 (38.5)	1.02 (0.49–2.10)	0.82 (0.41–1.66)
Caregiver financial difficulty			
No	38 (36.2)	1.00	1.00
Yes	9 (47.4)	1.31 (0.76–2.24)	1.29 (0.79–2.09)
*Travel-related factors*			
Long distance to clinic			
No	19 (40.4)	1.00	1.00
Yes	28 (36.4)	0.90 (0.57–1.42)	1.00 (0.61–1.64)
Transport fee is expensive			
No	30 (35.7)	1.00	1.00
Yes	17 (42.5)	1.19 (0.75–1.89)	1.30 (0.79–2.13)
Not enough time for visits			
No	38 (35.5)	1.00	1.00
Yes	9 (52.9)	1.49 (0.89–2.50)	1.27 (0.78–2.09)
*Psychosocial factors*			
Visits noticed by community			
No	38 (39.2)	1.00	1.00
Yes	9 (33.3)	0.85 (0.47–1.53)	0.75 (0.41–1.39)
Visits noticed by school/peer			
No	29 (34.5)	1.00	1.00
Yes	18 (45.0)	1.30 (0.83–2.05)	1.21 (0.76–1.93)
Distrust health care workers			
No	45 (38.5)	1.00	1.00
Yes	2 (28.6)	0.74 (0.23–2.45)	0.84 (0.25–2.76)
Disclose status to family			
No	3 (50.0)	1.00	1.00
Yes	44 (37.9)	0.76 (0.33–1.75)	0.62 (0.26–1.50)
Disclose status to school/peer			
No	34 (41.0)	1.00	1.00
Yes	13 (32.5)	0.79 (0.47–1.33)	0.66 (0.40–1.09)
*Health care facility factors*			
Long waiting queues at clinic			
No	38 (43.7)	1.00	1.00
Yes	9 (24.3)	0.56 (0.30–1.03)	0.54 (0.30–0.99)
Inconvenient clinic hours			
No	38 (36.9)	1.00	1.00
Yes	9 (42.9)	1.16 (0.67–2.02)	1.20 (0.70–2.05)
Unfriendly health care workers			
No	44 (36.7)	1.00	1.00
Yes	3 (75.0)	2.05 (1.11–3.77)	1.61 (0.81–3.17)
No sexual health services			
No	41 (37.3)	1.00	1.00
Yes	6 (42.9)	1.15 (0.60–2.21)	1.26 (0.69–2.30)
Lack of peer support			
No	41 (36.9)	1.00	1.00
Yes	6 (46.2)	1.25 (0.66–2.36)	0.93 (0.48–1.82)
*Treatment-related factors*			
Having problems taking ART			
No	33 (33.0)	1.00	1.00
Yes	14 (58.3)	1.77 (1.14–2.74)	1.45 (0.91–2.32)
Treatment fatigue			
No	32 (32.7)	1.00	1.00
Yes	15 (57.7)	1.77 (1.14–2.73)	1.47 (0.94–2.31)

^*∗*^Risk ratios individually adjusted for age, gender, mother as caregiver, and time on ART.

**Table 4:** Factors associated with prevalent virologic failure among adolescents with a recorded VL measurement (*n* = 103).

Variable	*n* (%) with prevalent VL failure	Crude risk ratio (95% CI)	Adjusted^*∗*^risk ratio (95% CI)
*Baseline and demographic factors*			
Gender			
Female	16 (27.1%)	1.00	1.00
Male	16 (36.4%)	1.54 (0.66–3.56)	1.44 (0.76–2.73)
Age group (years)			
12–14	11 (25.6%)	1.00	1.00
15–17	11 (37.9%)	1.78 (0.64–4.91)	2.67 (0.76–9.34)
18–20	10 (32.3%)	1.39 (0.50–3.83)	2.77 (0.51–14.96)
Highest school level			
Secondary	14 (35.0%)	1.00	1.00
Primary	14 (25.9%)	0.65 (0.27–1.58)	1.17 (0.28–4.79)
Dwelling type			
Formal	26 (32.9%)	1.00	—
Care facility	1 (9.1%)	0.20 (0.02–1.68)	—
Informal	5 (38.5%)	1.27 (0.38–4.28)	—
Mother caregiver			
No	15 (31.3%)	1.00	—
Yes	17 (30.9%)	0.98 (0.43–2.27)	—
Caregiver employed			
No	10 (35.7%)	1.00	—
Yes	21 (32.3%)	0.86 (0.34–2.18)	—
*Caregiver-related factors*			
Caregiver changes frequently			
No	32 (32.7%)	1.00	—
Yes	0 (0.0%)	—	—
Caregiver is elderly			
No	22 (30.6%)	1.00	1.00
Yes	10 (32.3%)	1.08 (0.44–2.67)	1.31 (0.69–2.48)
Caregiver is ill, requires care			
No	29 (29.9%)	1.00	1.00
Yes	3 (50.0%)	2.34 (0.45–12.31)	0.65 (0.09–4.55)
Caregiver is unsupportive			
No	28 (30.8%)	1.00	—
Yes	4 (33.3%)	1.13 (0.31–4.05)	—
Caregiver financial difficulty			
No	26 (30.2%)	1.00	1.00
Yes	6 (35.3%)	1.26 (0.42–3.77)	2.51 (0.51–12.38)
*Travel-related factors*			
Long distance to clinic			
No	11 (28.2%)	1.00	1.00
Yes	21 (32.8%)	1.24 (0.52–2.97)	0.43 (0.18–1.04)
Transport fee is expensive			
No	20 (27.8%)	1.00	1.00
Yes	12 (38.7%)	1.64 (0.68–3.99)	1.79 (0.76–4.19)
Not enough time for visits			
No	28 (31.1%)	1.00	—
Yes	4 (30.8%)	0.98 (0.28–3.47)	—
*Psychosocial factors*			
Visits noticed by community			
No	32.9% (26)	1.00	1.00
Yes	25.0% (6)	0.68 (0.24–1.92)	0.37 (0.15–0.93)
Visits noticed by school/peer			
No	31.4% (22)	1.00	1.00
Yes	30.3% (10)	0.95 (0.39–2.33)	1.02 (0.44–2.37)
Distrust health care workers			
No	30.3% (30)	1.00	1.00
Yes	50.0% (2)	2.30 (0.31–17.10)	6.15 (1.14–33.24)
Disclose status to family			
No	33.3% (1)	1.00	1.00
Yes	31.3% (31)	0.91 (0.08–10.44)	1.34 (0.39–4.63)
Disclose status to school/peer			
No	34.3% (24)	1.00	1.00
Yes	25.0% (8)	0.64 (0.25-1.64)	0.51 (0.20–1.26)
*Health care facility factors*			
Long waiting queues at clinic			
No	27 (35.5%)	1.00	1.00
Yes	5 (18.5%)	0.41 (0.14–1.21)	0.94 (0.28–3.12)
Inconvenient clinic operating hours			
No	28 (32.6%)	1.00	1.00
Yes	4 (23.5%)	0.64 (0.19–2.13)	0.49 (0.16–1.52)
Unfriendly health care workers			
No	32 (31.4%)	1.00	—
Yes	0 (0.0%)	—	—
No sexual health services			
No	28 (31.1%)	1.00	1.00
Yes	4 (30.8%)	0.98 (0.28–3.47)	0.70 (0.19–2.50)
Lack of peer support			
No	31 (33.0%)	1.00	—
Yes	1 (11.1%)	0.25 (0.03–2.12)	—
*Treatment-related factors*			
Having problems taking ART			
No	26 (31.3%)	1.00	1.00
Yes	6 (30.0%)	0.94 (0.32–2.72)	0.36 (0.16–0.82)
Treatment fatigue			
No	23 (28.4%)	1.00	1.00
Yes	9 (40.9%)	1.75 (0.66–4.64)	1.71 (0.78–3.75)

^*∗*^Risk ratios individually adjusted for age, gender, mother as caregiver, and time on ART.
