# Supplementary material for: Insights into Adherence among a Cohort of Adolescents Aged 12–20 Years in South Africa: Reported Barriers to Antiretroviral Treatment
Source: AIDS Res Treat. 2016 Oct 27;2016:4161738. doi: 10.1155/2016/4161738 (PMC5102702; doi:10.1155/2016/4161738)
Supplement: Supplementary file 1 — The supplementary material presents the analysis of risk factors associated with having at least one late or missed visit stratified by age category. First, we estimate factors associated with the primary outcome for younger adolescents aged 12-17 years (Supplementary Table A) and second, for older adolescents aged 18-20 years (Supplementary Table B). [file 4161738.f1.docx]

**Supplementary Table A: Factors associated with having at least one late or missed visit within adolescents aged 12-17 years (n=88)**

| **Characteristic** |  | **N (%) with missed visit** | **Crude**  **risk ratio**  **(95% CI)** | **Adjusted**  **risk ratio***  **(95% CI)** |
| --- | --- | --- | --- | --- |
| ***Baseline and demographic factors*** |  |  |  |  |
| Gender; n (%) | Female | 16 (37.2) | 1.00 | 1.00 |
|  | Male | 13 (29.5) | 0.79 (0.44-1.45) | 0.74 (0.41-1.34) |
| Highest school level; n (%) | Secondary | 8 (42.1) | 1.00 | 1.00 |
|  | Primary | 20 (30.8) | 0.73 (0.38-1.39) | 0.84 (0.44-1.62) |
| Dwelling type; n (%) | Formal | 26 (38.2) | 1.00 | 1.00 |
|  | Care facility | 1 (14.3) | 0.37 (0.06-2.35) | 0.28 (0.04-1.80) |
|  | Informal | 2 (16.7) | 0.44 (0.12-1.60) | 0.60 (0.15-2.32) |
| Mother as caregiver; n (%) | No | 16 (42.1) | 1.00 | 1.00 |
|  | Yes | 13 (26.5) | 0.63 (0.35-1.14) | 0.60 (0.33-1.10) |
| Caregiver employed; n (%) | No | 7 (29.2) | 1.00 | 1.00 |
|  | Yes | 21 (37.5) | 1.29 (0.63-2.61) | 1.55 (0.75-3.20) |
|  | Not applicable | 1 (14.3) | 0.49 (0.07-3.34) | 0.37 (0.05-2.49) |
| Time on ART | <6 years | 13 (32.5) | 1.00 | 1.00 |
|  | >6 years | 16 (34.0) | 1.05 (0.58-1.91) | 1.31 (0.38-4.58) |
| ***Caregiver-related factors*** |  |  |  |  |
| Caregiver changes frequently; n (%) | No | 27 (33.3) | 1.00 | 1.00 |
|  | Yes | 2 (33.3) | 1.00 (0.31-3.23) | 0.85 (0.26-2.80) |
| Caregiver is elderly; n (%) | No | 20 (32.3) | 1.00 | 1.00 |
|  | Yes | 9 (36.0) | 1.12 (0.59-2.11) | 0.93 (0.49-1.78) |
| Caregiver is ill and requires care; n (%) | No | 28 (34.1) | 1.00 | 1.00 |
|  | Yes | 1 (20.0) | 0.59 (0.10-3.47) | 0.74 (0.12-4.50) |
| Caregiver is unsupportive; n (%) | No | 27 (32.9) | 1.00 | 1.00 |
|  | Yes | 2 (40.0) | 1.21 (0.40-3.71) | 1.76 (0.52-5.94) |
| Caregiver has financial difficulty; n (%) | No | 25 (33.3) | 1.00 | 1.00 |
|  | Yes | 4 (33.3) | 1.00 (0.42-2.37) | 1.23 (0.50-3.05) |
| ***Travel-related factors*** |  |  |  |  |
| Long distance to clinic; n (%) | No | 11 (35.5) | 1.00 | 1.00 |
|  | Yes | 18 (32.1) | 0.91 (0.49-1.66) | 0.94 (0.51-1.70) |
| Transport fee is expensive; n (%) | No | 17 (28.8) | 1.00 | 1.00 |
|  | Yes | 12 (42.9) | 1.49 (0.83-2.67) | 1.74 (0.95-3.19) |
| Not enough time for visits; n (%) | No | 23 (30.3) | 1.00 | 1.00 |
|  | Yes | 6 (54.5) | 1.80 (0.95-3.41) | 1.97 (1.10-3.51) |
| ***Psychosocial factors*** |  |  |  |  |
| Visits noticed by family/community; n (%) | No | 24 (33.8) | 1.00 | 1.00 |
|  | Yes | 5 (31.3) | 0.92 (0.42-2.05) | 1.25 (0.52-2.99) |
| Visits noticed by friends/school; n (%) | No | 16 (27.1) | 1.00 | 1.00 |
|  | Yes | 13 (46.4) | 1.71 (0.96-3.05) | 1.80 (1.01-3.21) |
| Distrust health care workers; n (%) | No | 27 (33.8) | 1.00 | 1.00 |
|  | Yes | 2 (28.6) | 0.85 (0.25-2.84) | 0.81 (0.25-2.64) |
| Disclosed status to family; n (%) | No | 3 (50.0) | 1.00 | 1.00 |
|  | Yes | 26 (32.5) | 0.65 (0.27-1.54) | 0.45 (0.18-1.17) |
| Disclosed status to school/friends; n (%) | No | 22 (34.9) | 1.00 | 1.00 |
|  | Yes | 7 (30.4) | 0.87 (0.43-1.76) | 0.89 (0.44-1.80) |
| ***Healthcare facility factors*** |  |  |  |  |
| Long waiting queues at clinic; n (%) | No | 23 (37.7) | 1.00 | 1.00 |
|  | Yes | 6 (23.1) | 0.61 (0.28-1.32) | 0.67 (0.31-1.47) |
| Inconvenient clinic operating hours; n (%) | No | 23 (31.9) | 1.00 | 1.00 |
|  | Yes | 6 (40.0) | 1.25 (0.62-2.54) | 1.99 (0.79-4.99) |
| Unfriendly health care workers; n (%) | No | 28 (32.9) | 1.00 | 1.00 |
|  | Yes | 1 (50.0) | 1.52 (0.37-6.27) | 1.42 (0.32-6.31) |
| No sexual health services; n (%) | No | 25 (32.9) | 1.00 | 1.00 |
|  | Yes | 4 (36.4) | 1.11 (0.47-2.57) | 1.24 (0.53-2.90) |
| Lack of peer support/counselling; n (%) | No | 25 (31.3) | 1.00 | 1.00 |
|  | Yes | 4 (57.1) | 1.83 (0.89-3.75) | 1.74 (0.91-3.33) |
| ***Treatment-related factors*** |  |  |  |  |
| Having problems taking ART; n (%) | No | 22 (29.7) | 1.00 | 1.00 |
|  | Yes | 7 (53.8) | 1.81 (0.98-3.34) | 2.02 (1.05-3.88) |
| Treatment fatigue; n (%) | No | 21 (29.2) | 1.00 | 1.00 |
|  | Yes | 8 (53.3) | 1.83 (1.01-3.31) | 1.76 (0.98-3.16) |

* Risk ratios individually adjusted for age, gender, mother as caregiver and time on ART

**Supplementary Table B: Factors associated with having at least one late or missed visit within adolescents aged 18-20 years (n=38)**

| **Characteristic** |  | **N (%) with missed visit** | **Crude**  **risk ratio**  **(95% CI)** | **Adjusted**  **risk ratio***  **(95% CI)** |
| --- | --- | --- | --- | --- |
| ***Baseline and demographic factors*** |  |  |  |  |
| Gender; n (%) | Female | 11 (42.3) | 1.00 | 1.00 |
|  | Male | 7 (63.6) | 1.50 (0.80-2.83) | 1.51 (0.83-2.74) |
| Highest school level; n (%) | Secondary | 13 (46.4) | 1.00 | 1.00 |
|  | Primary | 2 (100) | 2.15 (1.45-3.21) | 2.01 (0.69-5.91) |
| Dwelling type; n (%) | Formal | 10 (38.5) | 1.00 | 1.00 |
|  | Care facility | 4 (66.7) | 1.73 (0.82-3.65) | 4.80 (1.35-17.05) |
|  | Informal | 4 (43.8) | 2.08 (1.08-4.00) | 4.25 (1.80-10.05) |
| Mother as caregiver; n (%) | No | 11 (52.4) | 1.00 | 1.00 |
|  | Yes | 7 (43.8) | 0.84 (0.42-1.66) | 0.70 (0.37-1.34) |
| Caregiver employed; n (%) | No | 3 (33.3) | 1.00 | 1.00 |
|  | Yes | 11 (47.8) | 1.43 (0.52-3.97) | 1.41 (0.56-3.54) |
|  | Not applicable | 4 (80.0) | 2.40 (0.86-6.67) | 3.71 (0.99-13.95) |
| Time on ART | <6 years | 11 (55.0) | 1.00 | 1.00 |
|  | >6 years | 7 (41.2) | 0.75 (0.37-1.50) | 0.89 (0.25-3.14) |
| ***Caregiver-related factors*** |  |  |  |  |
| Caregiver changes frequently; n (%) | No | 18 (51.4) |  |  |
|  | Yes | 0 (0.0) | - |  |
| Caregiver is elderly; n (%) | No | 10 (45.5) | 1.00 | 1.00 |
|  | Yes | 8 (53.3) | 1.17 (0.61-2.27) | 1.05 (0.56-1.96) |
| Caregiver is ill and requires care; n (%) | No | 16 (47.1) | 1.00 | 1.00 |
|  | Yes | 2 (66.7) | 1.42 (0.59-3.40) | 1.07 (0.44-2.57) |
| Caregiver is unsupportive; n (%) | No | 15 (51.7) | 1.00 | 1.00 |
|  | Yes | 3 (37.5) | 0.73 (0.28-1.90) | 0.69 (0.28-1.71) |
| Caregiver financial difficulty; n (%) | No | 13 (43.3) | 1.00 | 1.00 |
|  | Yes | 5 (71.4) | 1.65 (0.88-3.07) | 1.51 (0.77-2.98) |
| ***Travel-related factors*** |  |  |  |  |
| Long distance to clinic; n (%) | No | 8 (50.0) | 1.00 | 1.00 |
|  | Yes | 10 (47.6) | 0.95 (0.49-1.85) | 0.95 (0.49-1.83) |
| Transport fee is expensive; n (%) | No | 13 (52.0) | 1.00 | 1.00 |
|  | Yes | 5 (41.7) | 0.80 (0.37-1.73) | 0.83 (0.40-1.74) |
| Not enough time for visits; n (%) | No | 15 (48.4) | 1.00 | 1.00 |
|  | Yes | 3 (50.0) | 1.03 (0.43-2.49) | 0.93 (0.39-2.27) |
| ***Psychosocial factors*** |  |  |  |  |
| Visits noticed by family/community; n (%) | No | 14 (53.8) | 1.00 | 1.00 |
|  | Yes | 4 (36.4) | 0.68 (0.29-1.59) | 0.59 (0.26-1.35) |
| Visits noticed by friends/school; n (%) | No | 13 (52.0) | 1.00 | 1.00 |
|  | Yes | 5 (41.7) | 0.80 (0.37-1.73) | 0.74 (0.35-1.58) |
| Distrust health care workers; n (%) | No | 18 (48.6) | - | - |
|  | Yes | 0 (0.0) | - | - |
| Disclosed status to family; n (%) | No | 0 (0.0) | - | - |
|  | Yes | 18 (50.0) | - | - |
| Disclosed status to school/friends; n (%) | No | 12 (60.0) | 1.00 | 1.00 |
|  | Yes | 6 (35.3) | 0.59 (0.28-1.23) | 0.38 (0.15-0.95) |
| ***Healthcare facility factors*** |  |  |  |  |
| Long waiting queues at clinic; n (%) | No | 15 (57.7) | 1.00 | 1.00 |
|  | Yes | 3 (27.3) | 0.47 (0.17-1.31) | 0.38 (0.14-1.02) |
| Inconvenient clinic operating hours; n (%) | No | 15 (48.4) | 1.00 | 1.00 |
|  | Yes | 3 (50.0) | 1.03 (0.43-2.49) | 1.15 (0.42-3.11) |
| Unfriendly health care workers; n (%) | No | 16 (45.7) | 1.00 | 1.00 |
|  | Yes | 2 (100.0) | 2.19 (1.52-3.14) | 1.38 (0.69-2.77) |
| No sexual health services; n (%) | No | 16 (47.1) | 1.00 | 1.00 |
|  | Yes | 2 (66.7) | 1.42 (0.59-3.40) | 1.42 (0.62-3.27) |
| Lack of peer support/counselling; n (%) | No | 16 (51.6) | 1.00 | 1.00 |
|  | Yes | 2 (33.3) | 0.65 (0.20-2.11) | 0.60 (0.17-2.15) |
| ***Treatment-related factors*** |  |  |  |  |
| Having problems taking ART; n (%) | No | 11 (42.3) | 1.00 | 1.00 |
|  | Yes | 7 (63.6) | 1.50 (0.80-2.83) | 1.59 (0.78-3.25) |
| Treatment fatigue; n (%) | No | 11 (42.3) | 1.00 | 1.00 |
|  | Yes | 7 (63.6) | 1.50 (0.80-2.83) | 1.58 (0.81-3.07) |

* Risk ratios individually adjusted for age, gender, mother as caregiver and time on ART
